# Regulation of ectodomain shedding of ADAM33 *in vitro* and *in vivo*

**DOI:** 10.1016/j.jaci.2019.01.026

**Published:** 2019-06

**Authors:** Elizabeth R. Davies, Laura Denney, Marieke Wandel, Clare M. Lloyd, Donna E. Davies, Hans Michael Haitchi

**Affiliations:** a Brooke Laboratories, Clinical and Experimental Sciences, Faculty of Medicine, University of Southampton, Southampton, United Kingdom; b Inflammation, Repair & Development, National Heart and Lung Institute, Imperial College London, London, United Kingdom; c National Institute for Health Research Southampton Biomedical Research Centre at University Hospital Southampton NHS Foundation Trust, Southampton, United Kingdom; d Institute for Life Sciences, University of Southampton, Southampton, United Kingdom

To the Editor:

A disintegrin and metalloprotease *(ADAM)33* is a susceptibility gene for asthma and bronchial hyperresponsiveness (BHR). ADAM33 is a transmembrane protein, but a soluble protein containing the metalloprotease domain (sADAM33) has previously been identified in bronchoalveolar lavage fluid (BALF) from subjects with asthma and its levels significantly and negatively correlated with FEV_1_, suggesting a role in the development of airflow obstruction.^1^ We have shown that sADAM33 in asthmatic BALF is enzymatically active and that the sADAM33 metalloprotease causes angiogenesis and myogenesis *in vivo* or *ex vivo*.^2^, ^3^ Furthermore, *in vivo* allergen challenge causes shedding of enzymatically active sADAM33 into BALF of wild-type mice, and in a transgenic mouse model, lung-specific sADAM33 expression causes airway remodeling, which enhances eosinophil recruitment with associated BHR following allergen sensitization and challenge.^2^ Although TGF-β is a trigger for sADAM33 release *in vitro*,^3^ there is no mechanistic information or evidence of *in vivo* relevance. Because it is not possible to test directly the importance of TGF-β in ectodomain shedding in human asthma, we characterized the mechanism(s) of the TGF-β–induced ectodomain shedding of murine ADAM33 and determined its importance for shedding of ADAM33 *in vivo.* Detailed methodology is provided in the Methods section in this article's Online Repository at www.jacionline.org.

Initially, we confirmed that murine ADAM33 was similar to human ADAM33 in its sensitivity to TGF-β–induced ectodomain shedding.^3^ As expected, TGF-β treatment caused a dose-dependent increase in sADAM33 in supernatants of Cos-7 cells expressing murine ADAM33 (see Fig E1, *A* and *B*, in this article's Online Repository at www.jacionline.org). The main band had a molecular weight of around 102 kDa, indicating that the entire ectodomain had been shed; however, further processing was also evident, suggesting loss of the inhibitory prodomain. Consistent with this, there was a significant increase in ADAM33 enzymatic activity in cell-free supernatants following TGF-β treatment (Fig E1, *C*). Pericellular proteolysis is frequently mediated by members of the ADAM or MT-MMP families that are sensitive to the broad-spectrum hydroxamic acid–based inhibitor, GM6001; however, we found that it did not affect the shedding (see Fig E2, *A* and *B*, in this article's Online Repository at www.jacionline.org) or activity (Fig E2, *C*) of ADAM33. We also confirmed that GM6001 had no effect on the activity of purified recombinant ADAM33 (Fig E2, *D*). Because ADAM33 has a unique substrate-binding site and its catalytic activity is insensitive to GM6001,^4^ we postulated that ADAM33 ectodomain shedding was autocatalytic. Consistent with this, mutation of E347A in the catalytic site suppressed shedding of sADAM33 both at baseline and in response to TGF-β (Fig 1, *A*) and this was accompanied by a significant reduction in enzymatic activity in cell-free supernatants (Fig 1, *B*). These data suggest that a substantial component of the shedding of sADAM33 is autocatalytic.

**Fig 1:**
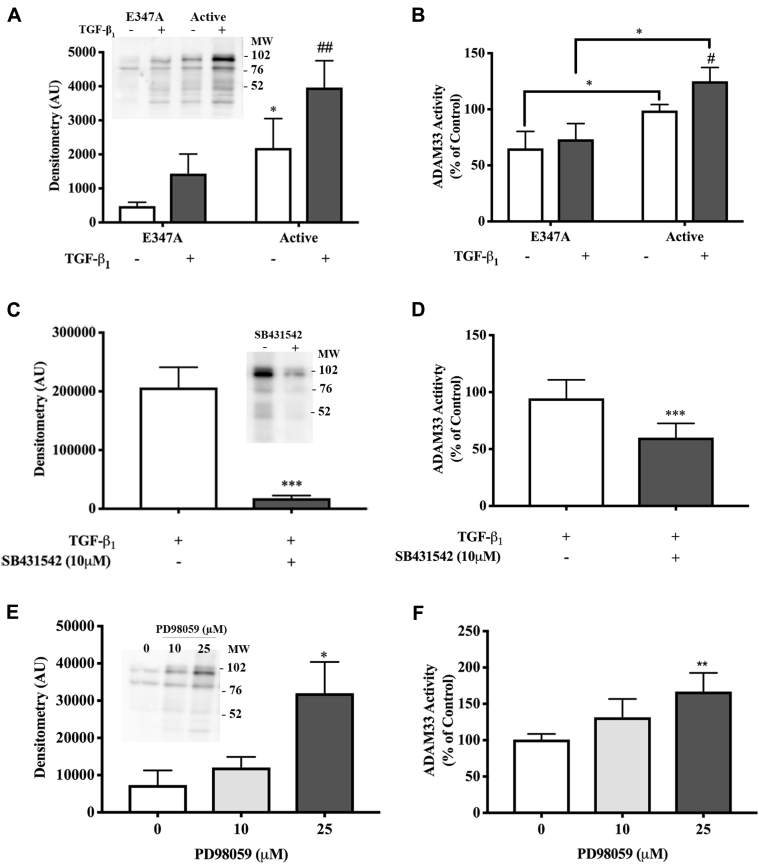
Regulation of ADAM33 shedding. Cos-7 cells expressing full-length catalytically active murine ADAM33 **(A-F)** or inactive ADAM33 E347A (Fig 1, *A* and *B*) were treated without or with TGF-β (5 ng/mL) for 8 hours and SMAD (SB431542) (Fig 1, *C* and *D*) or MAPK (PD98059) (Fig 1, *E* and *F*) inhibitors, as indicated. Soluble ADAM33 immunoreactivity and enzymatic activity in cell-free supernatants were assessed by Western blotting (inset) with densitometry (arbitrary units, AU) (Fig 1, *A*, *C*, and *E*) or in a fluorescence resonance energy transfer (FRET) peptide cleavage assay, respectively; in panel Fig 1, *B*, enzymatic activity in the supernatants is expressed as a percent of that measured in supernatants from untreated cells expressing full-length active ADAM33, whereas in panels *D* and *F*, activity is expressed as a percent of that measured in supernatants from TGF-β–treated cells. Data are expressed as mean + SD (n = 4 independent experiments). #*P* < .05, ##*P* < .01 vs control, *P < .05, **P < .01, ***P < .001.

TGF-β activates multiple signals including mothers against decapentaplegic homolog (SMAD) and non-SMAD pathways. Inhibition of SMAD signaling using SB431452 significantly suppressed TGF-β_1_–induced shedding of immunoreactive and enzymatically active ADAM33 (Fig 1, *C* and *D*). In contrast, the MAP2K/mitogen-activated protein kinase kinase inhibitor PD98059 dose-dependently increased shedding of immunoreactive and enzymatically active forms of sADAM33 (Fig 1, *E* and *F*). These data suggest a complex effect of TGF-β signaling, with SMAD activation being stimulatory for ADAM33 shedding, whereas mitogen-activated protein kinase (MAPK) activation has a negative regulatory effect. The latter effect may be mediated via TIMP-3, a known inhibitor of ADAM33 enzymatic activity,^5^ which is reported to be induced by TGF-β via MAPK signaling.^6^ Thus, inhibiting MAPK activation with PD98059 may inhibit TIMP3 expression and release ADAM33 from the effects of this natural inhibitor leading to increased autocatalytic shedding and augmented enzyme activity. Although ectodomain shedding can be regulated by natural protein inhibitors, other mechanisms including membrane trafficking, protein maturation, and substrate presentation have also been shown to be important for the regulation of other sheddases.^7^ Whether release of ADAM33 is preceded by its membrane trafficking or maturation and/or by assembly into higher order complexes requiring additional protein interactions to exosites located either on the ADAM33 or adapter proteins remains to be determined.

Epithelial injury leads to release of TGF-β, and we have postulated that this is the source of the TGF-β that drives the increase in sADAM33 observed in response to allergen challenge *in vivo*.^2^ To test this hypothesis, we used BALF from mice in which lung epithelial *Tgfb1* was conditionally deleted in bronchial epithelial club cells before intranasal administration of either 25 μg house dust mite extract or recombinant murine IL-33.^8^ After house dust mite challenge, lower levels of sADAM33 could be detected in the BALF of *Tgfb1*^*−/−*^ mice compared with littermate controls (Fig 2, *A*) and it also contained less sADAM33 enzymatic activity (Fig 2, *B*). In the same way, when mice were challenged with IL-33, BALF from *Tgfb1*^*−/−*^ mice had a lower level of sADAM33 immunoreactive protein (Fig 2, *C*) and enzymatic activity (Fig 2, *D*). Of note, exogenous TGF-β alone was ineffective at stimulating shedding of ADAM33 *in vivo*. This might be explained either by the fact that TGF-β could not pass through the intact epithelium of unchallenged mice and/or by the low dose and short half-life of the growth factor, which did not allow sufficient time for it to pass through the epithelium to reach the subepithelial mesenchymal cells where ADAM33 is expressed.^9^

**Fig 2:**
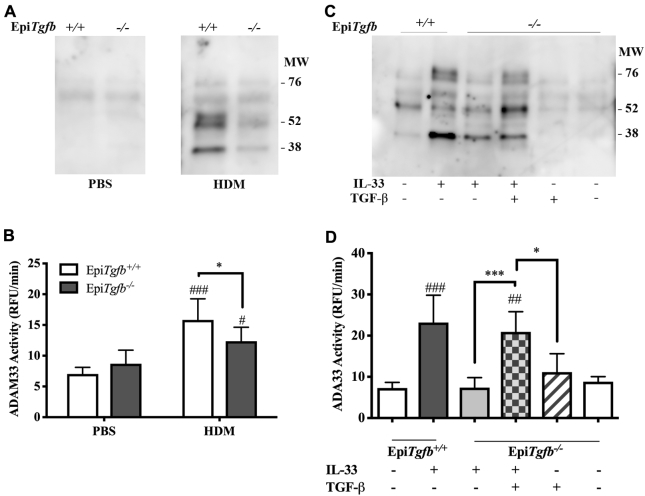
Epithelial TGF-β enhances shedding of ADAM33 *in vivo.* Epithelial (Epi)*Tgfb*^*−/−*^ or littermate *Tgfb*^*+/+*^ control mice were challenged with intranasal house dust mite (HDM) extract (**A** and **B**) or recombinant IL-33 (**C** and **D**) and, where indicated, recombinant TGF-β (Fig 2, *C* and *D*). Soluble ADAM33 immunoreactivity and enzyme activity in BALF were assessed by Western blotting (Fig 2, *A* and *C*) or FRET peptide cleavage assay, respectively; enzyme activity is expressed as relative fluorescent units per minute (RFU/min) (Fig 2, *B* and *D*). *FRET*, Fluorescence Resonance Energy Transfer. Data are mean + SD (n = 4-6 mice per group). Data are representative of 2 independent experiments. ##*P* < .01, ###*P* < .001 vs control, *P < .05, ***P < .001.

Epithelial-derived IL-33 induces rapid release of TGF-β into the airways to enhance migration of innate lymphoid cells (ILCs) and development of a robust ILC2 response that initiates allergic immunity.^9^ Because we found that epithelial-derived TGF-β is also required for ectodomain shedding of ADAM33, it may be speculated that sADAM33 can contribute to the recruitment or activation of ILCs or other innate immune cells, either by affecting matrix turnover or by promoting growth factor or chemokine shedding. Of note, polymorphisms in *TGFB*, *IL33*, and *ADAM33* genes have each been associated with asthma susceptibility, yet each has a small overall effect on disease development. The involvement of 3 susceptibility gene products in epithelial responses to allergens highlights how they may cooperate to amplify the downstream asthmatic responses.

Identification of the involvement of TGF-β in ectodomain shedding of ADAM33 in an *in vivo* model strengthens the case for exploring how human polymorphic variation in the *ADAM33* gene is linked to asthma pathogenesis. Four single nucleotide polymorphisms (S1, S2, T1, and T2) encode amino acid substitutions in the transmembrane and cytoplasmic domain of ADAM33 and have been associated with asthma.^4^ Although the intracellular domain of ADAM33 is relatively short, it is very rich in prolines, having a putative SH3 binding site where the T2 SNP is located, a casein kinase I/II phosphorylation site, and an MAPK consensus sequence that is likely to be important for regulation of ADAM33 function, especially as we have identified a negative regulatory role for MAPK in our current studies. Further work is required to determine whether this effect is direct and involves ADAM33 phosphorylation or indirect via inhibitors such as TIMP3. Alternatively, one mutation Ala395Val is located within the catalytic domain,^4^ which may directly affect catalytic activity.

In summary, we have provided direct evidence that epithelial-derived TGF-β is an important regulator of ectodomain shedding of enzymatically active ADAM33 from the mesenchyme. This process appears largely to be autocatalytic and involves SMAD signaling, but is negatively regulated by MAPK signaling. These findings highlight the importance of epithelial-mesenchymal cross-talk in asthma pathogenesis and underscore the potential for co-operation between different asthma susceptibility genes to drive disease pathogenesis.

## Figures and Tables

**Fig E1:**
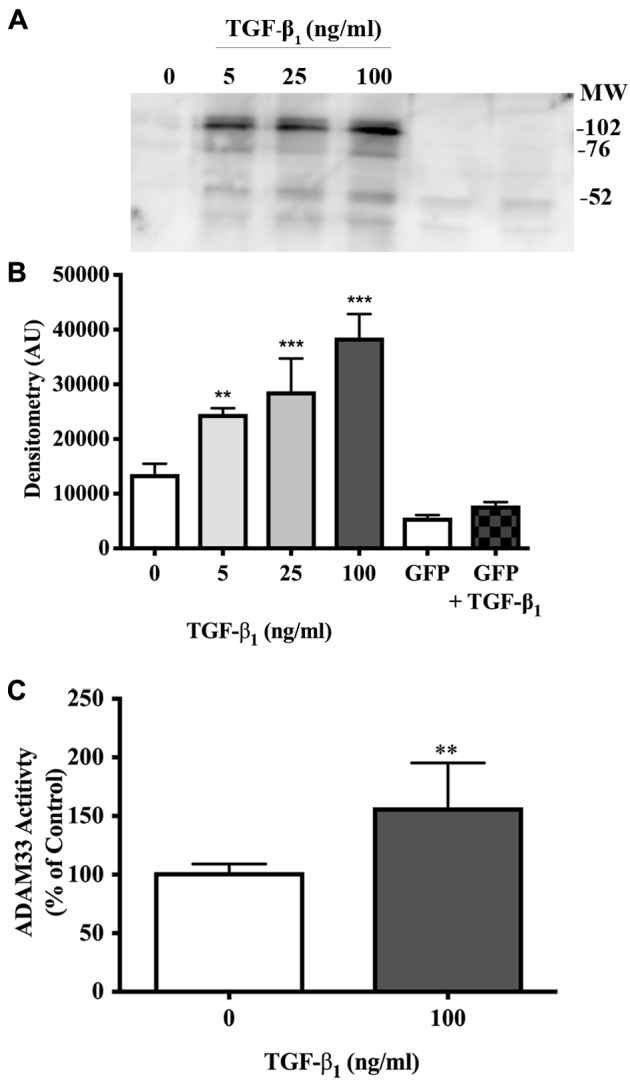
TGF-β enhances shedding of murine ADAM33. Cos-7 cells transiently transfected with full-length murine ADAM33 or green fluorescent protein (GFP) as control were treated with TGF-β as indicated. Soluble ADAM33 immunoreactivity and enzyme activity in cell-free supernatants were assessed by Western blotting **(A)** with densitometry (arbitrary units, AU) **(B)** or fluorescence resonance energy transfer peptide cleavage assay, respectively; enzyme activity is expressed as percent of activity found in supernatants from control untreated cells **(C)**. Data are mean + SD and are representative of 4 independent experiments. *MW*, Molecular weight. **P < .01. ***P < .001.

**Fig E2:**
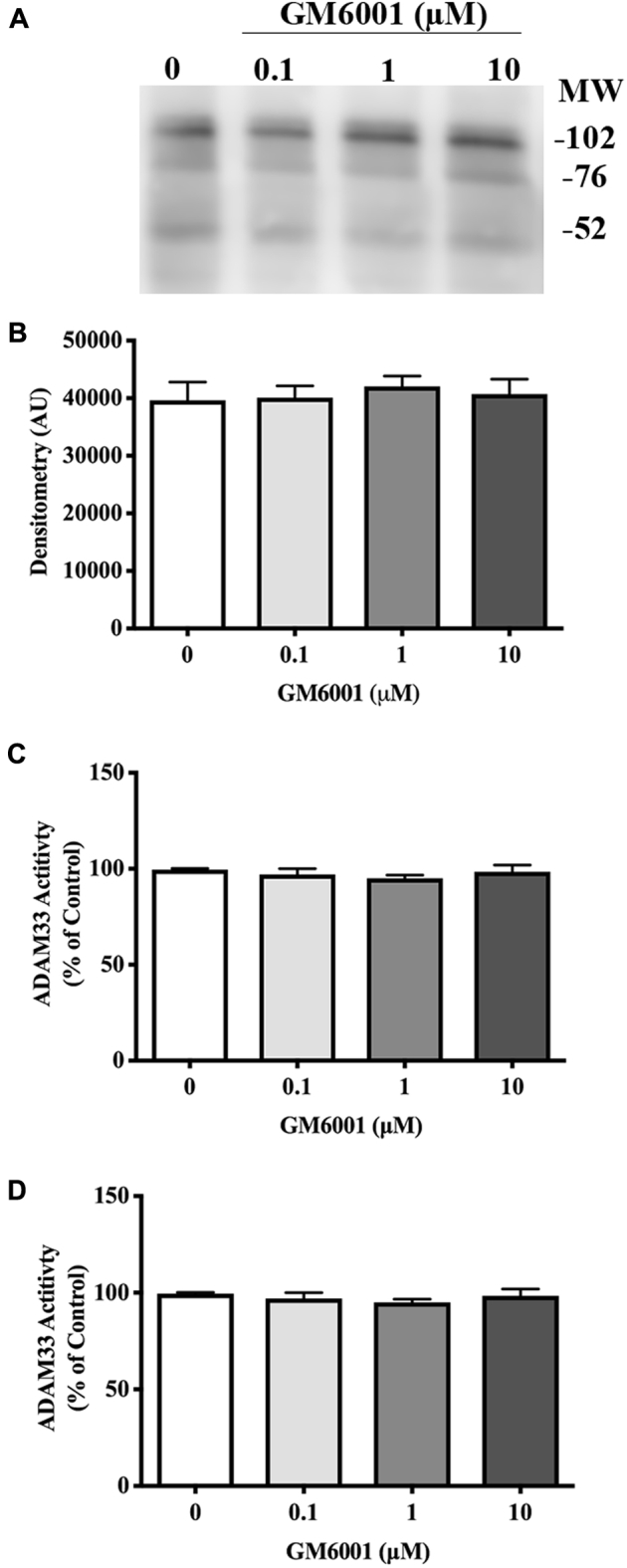
The broad-spectrum MMP inhibitor GM6001 does not inhibit ADAM33 shedding. Cos-7 cells transiently transfected with full-length murine ADAM33 were preincubated with GM6001 (0-10 μM) for 1 hour before addition of 5 ng/mL TGF-β for 8 hours. Cell-free supernatants were assessed for soluble ADAM33 by Western blotting **(A)** with densitometry (arbitrary units, AU) **(B)** and by fluorescence resonance energy transfer peptide cleavage assay **(C)**. Purified recombinant ADAM33 protein was incubated with GM6001 and tested for activity in a fluorescence resonance energy transfer peptide cleavage assay **(D)**. Activity is presented as a percent of that measured in the absence of GM6001. *MW*, Molecular weight. Data are expressed as mean + SD and are representative of 4 independent experiments.

## Methods

### Cell culture

The Cos-7 cell line, a fibroblast-like cell line, was grown in Dulbecco modified Eagle medium supplemented with 10% FBS, 50 units/mL penicillin, 50 μg/mL streptomycin, 2 mM l-glutamine, 1 mM sodium pyruvate, and 1× nonessential amino acids (Dulbecco modified Eagle medium/FBS) (all from Life Technologies, Paisley, UK). For transfection, cells were placed in non-supplemented Opti-MEM (Life Technologies).

### Transfection of full-length mouse ADAM33

Cos-7 cells were transfected with a plasmid encoding full-length murine *ADAM33* (MR217277 clone, NM_033615; OriGene, Rockville, Md) or green fluorescence protein using X-treme Gene 9 reagent (Roche, Southampton, UK). After 24 hours, cells were treated with TGF-β_1_ (Peprotech, London, UK) for 8 hours to assess ectodomain shedding of ADAM33. The TGF-β_1_ isoform was chosen, as this is the major isoform in adult mice.^E1^ Where indicated, cells were preincubated for 1 hour with the ALK5/SMAD inhibitor SB431542 (10 μM; Stratech, Ely, UK), the mitogen-activated protein kinase kinase (MAP2K, MEK) inhibitor PD98059 (10 or 25 μM, Sigma, Poole, UK), the broad-spectrum metalloprotease inhibitor GM6001 (10 μM; Merck, Millipore, Watford, UK), or vehicle control before addition of 5 ng/mL TGF-β_1_. Cell-free supernatants were harvested and cells were lysed into RIPA buffer.

### ADAM33 pull down with concanavalin A beads

Concanavalin A-Sepharose 4B beads (ConA) (Sigma) were used to pull down glycosylated ADAM33 from the supernatants of the transfected Cos-7 cells, as previously described.^E2^ In brief, 1 mL supernatant was adjusted to contain 20 mM Tris HCl, pH 7.4, 0.5 M NaCl, and protease inhibitors (Sigma) before addition of activated ConA beads (20 μL beads per mL sample). After binding overnight at 4°C, the beads were washed twice in 20 mM Tris HCl, pH 7.4, containing 0.5 M NaCl and then twice in 20 mM Tris HCl, pH 7.4, to remove excess salt. The bound protein was then solubilized into SDS sample buffer containing 0.5 M methyl α-d-mannopyranoside for SDS-PAGE and Western blotting.

### Fluorescence resonance energy transfer peptide cleavage assay

An ADAM33 enzymatic assay was performed using a fluorescence resonance energy transfer peptide cleavage assay as previously described.^E3^ All assay measurements were made at 37°C in real time using a Bio-Rad CFX96 machine for detection of carboxyfluorescein reporter dye (FAM) (450-490 nm excitation and 510-530 nm detection), allowing determination of initial rates. Each reaction contained 7 μL neat BALF or cell supernatant mixed with 2 μL 5x-assay buffer (100 mM HEPES, pH 7, 2.5 M NaCl, 50 mM CaCl_2_, and 1 mg/mL BSA); the assay was initiated by addition of 44 pmol fluorescence resonance energy transfer peptide (DABCYL-YRVAFQKLAE(FAM)K-NH_2_) (Severn Biotech, Kidderminster, UK) and 100 pmol ZnCl_2_ to make a final reaction volume of 10 μL. Enzymatic activity was determined by plotting relative fluorescence units against time after the background had been subtracted. The rate of the reaction (relative fluorescence units/min) was determined from the line of best fit in the linear phase of the assay. Control assays used human recombinant soluble active ADAM33 (positive control) and mutant ADAM33 (E365A) (negative control) as previously described.^E2^

### SDS-PAGE and western blotting

Solubilized samples from ConA pull down or murine BALF were run on 12.5% acrylamide or 8% to 20% gradient gels (Bio-Rad, Watford, UK) and transferred onto polyvinylidene fluoride (PVDF) membranes. The transferred protein was assessed by Ponceau staining to confirm equivalent protein loading before blocking using 2.5% BSA in TBS/Tween and Western blotting. Membranes were probed with a polyclonal goat antibody against the ectodomain of mouse ADAM33 (AF2434, 1:1000, R&D Systems, Abingdon, UK). Secondary antibody was rabbit antigoat IgG horseradish peroxidase antibody (Merck; 1:5000). The blots were visualized using enhanced chemiluminescence (Clarity ECL; Bio-Rad) with an Amersham Imager 600 (GE Healthcare, Buckinghamshire, UK); densitometric images were semiquantified using ImageJ software (National Institutes of Health, Wis) and data plotted as relative intensity.

### The conditional lung epithelial *Tgb1*^*−/−*^ mouse model

Mice conditionally lacking lung epithelial *Tgfb* expression (*Ccsp*-cre*Tgfb1*^−/−^) were generated as previously described.^E4^ Mice were pretreated with doxycycline (Sigma) (or vehicle as control) to delete *Tgfb1* from the bronchiolar epithelial cells 72 hours before intranasal administration of either 25 μg HDM extract (1 mg/mL protein solution dissolved in PBS) or 25 μL of PBS 5 times a week for 3 weeks. Mice were culled 4 hours after the final challenge. In other experiments, carrier-free recombinant murine IL-33 (1 μg/dose in 25 μL PBS) (eBioscience, Thermo Fisher Scientific, Hemel Hempstead, UK) was administered 3 times a week for 1 week and mice culled 18 hours after the final dose. Where required, 50 ng recombinant TGF-β_1_ in PBS (R&D Systems) was administered intranasally without or with IL-33. At the point of killing, BALF was collected by washing the airways 3 times with 400 μL PBS. After centrifugation to remove cells, supernatants were stored at −80°C. The protein content of the BALF was quantified (BCA Assay, Thermofisher, Hemel Hempstead, UK) and normalized before use in ADAM33 enzyme assays or solubilization in 2x Laemmli sample buffer (Bio-Rad) for analysis of sADAM33 protein by Western blotting. Mice of both sexes were used between age 7 and 12 weeks, housed in specific-pathogen-free conditions, and given food and water *ad libitum*. All procedures were conducted in accordance with the Animals (Scientific Procedures) Act 1986.

### Statistics

Normal distribution of the numeric data was evaluated, and appropriate parametric or nonparametric statistical tests applied. All data are parametric and plotted as mean with 1 SD. Statistical significance was assessed by using Student *t* test (unpaired data) with Welch correction if SDs were not equal for comparisons between 2 groups. For comparison of 3 or more groups, a 1-way ANOVA with Dunn's multiple comparison test was used. For comparison of 2 or more groups with 2 independent variables, a 2-way ANOVA with Tukey multiple comparison test was used (**P* < .05, ***P* < .01, ****P* < .001).
